# Mouse Cognition-Related Behavior in the Open-Field: Emergence of Places of Attraction

**DOI:** 10.1371/journal.pcbi.1000027

**Published:** 2008-02-29

**Authors:** Anna Dvorkin, Yoav Benjamini, Ilan Golani

**Affiliations:** 1 Department of Zoology, Tel Aviv University, Tel Aviv, Israel; 2 Department of Statistics and Operation Research, Tel Aviv University, Tel Aviv, Israel; University College London, United Kingdom

## Abstract

Spatial memory is often studied in the Morris Water Maze, where the animal's spatial orientation has been shown to be mainly shaped by distal visual cues. Cognition-related behavior has also been described along “well-trodden paths”—spatial habits established by animals in the wild and in captivity reflecting a form of spatial memory. In the present study we combine the study of Open Field behavior with the study of behavior on well-trodden paths, revealing a form of locational memory that appears to correlate with spatial memory. The tracked path of the mouse is used to examine the dynamics of visiting behavior to locations. A visit is defined as either progressing through a location or stopping there, where progressing and stopping are computationally defined. We then estimate the probability of stopping at a location as a function of the number of previous visits to that location, i.e., we measure the effect of visiting history to a location on stopping in it. This can be regarded as an estimate of the familiarity of the mouse with locations. The recently wild-derived inbred strain CZECHII shows the highest effect of visiting history on stopping, C57 inbred mice show a lower effect, and DBA mice show no effect. We employ a rarely used, bottom-to-top computational approach, starting from simple kinematics of movement and gradually building our way up until we end with (emergent) locational memory. The effect of visiting history to a location on stopping in it can be regarded as an estimate of the familiarity of the mouse with locations, implying memory of these locations. We show that the magnitude of this estimate is strain-specific, implying a genetic influence. The dynamics of this process reveal that locations along the mouse's trodden path gradually become places of attraction, where the mouse stops habitually.

## Author Summary

Spatially guided behavior and spatial memory are central subjects in behavioral neuroscience. Many tasks have been developed for laboratory investigations of these subjects since no single task can reveal their full richness. Here we turn to the simplest and oldest “task”, which involves no task at all: introducing a mouse into a large arena and tracking its free behavior. Traditionally, the test is used for studying emotionality and locomotor behavior, using simple summaries of the mouse's path such as its length and the percent of time spent away from walls. More sophisticated computational analysis of the dynamics of the path enables us to separate visiting behavior at locations into stops and passings. Using this distinction, the mouse's path reveals quantifiable locational memory: the mouse's decision to stop in a location is based on its visiting history there. In some strains of mice, the visited locations gradually become places of attraction where the mouse stops habitually. In other strains, the phenomenon is not evident at all. Such quantifiable characterization of locational memory now enables further exploration of the senses that mediate this type of memory and allows measurement and comparisons across mouse strains and across genetic and pharmacological preparations.

## Introduction

In the present study we ask how can a kinematic description of Open-Field behavior lead to an understanding of a mouse's higher cognitive functions. We use the organization of elementary patterns for revealing memory-related phenomena.

Low-level kinematic features such as the animal's instantaneous location and speed are extracted from the tracked paths by using special smoothing algorithms [1]. These have been used to statistically partition the mouse's trajectory into intrinsically defined segments of progression and of staying-in-place (stops, lingering episodes; [2]). In previous work on rats, examination of the spatial distribution of stops revealed the home base-the most preferred place in the environment [3]. The home-base is used by the animal as a reference around which it performs structured roundtrips [4],[5]. The home-base also exerts a constraint on the number of stops per roundtrip: the probability of returning to the home-base is an increasing function of the number of stops already performed by the animal in that roundtrip [6]. The home-base acts as an attractor in 2 ways: first, in the vast majority of cases the animal stops in this place upon visiting it, and second, within a roundtrip, this place exerts a gradually increasing attraction on the rat to return to it. Both forms of attraction imply recognition and memory of home-base location. In the present study, starting with the same trajectory data, we approach the issue of recognition and memory of places in a different way, by examining stopping behavior across all locations in the periphery of the open field.

We accomplish this aim by establishing the history of visits to locations all around the periphery of the arena, where visits are classified as stops or passings. We then determine whether the number of previous visits to a location affects the animal's decision to stop in it. An effect of visiting history on the probability of stopping would imply recognition and therefore locational memory.

We used two inbred strains commonly contrasted for their spatial memory—C57BL/6, which is considered to have good spatial memory, and DBA/2, whose performance is poor (e.g., [7]–[9]; see however [10]), and as a third strain, the recently wild-derived strain CZECHII whose spatial behavior might be less affected by domestication.

This study, which has been part of an ethological analysis of mouse exploratory behavior [11]–[14], provides a high throughput test for locational memory.

## Results

**Figure 1:**
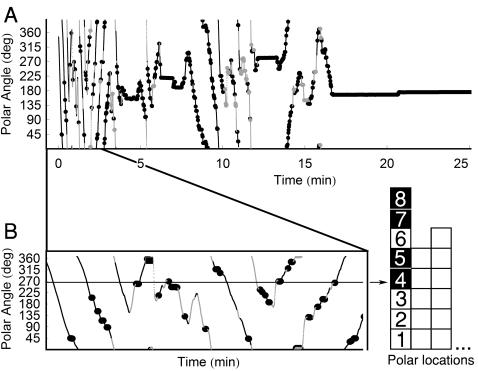
Establishing a Record of Visits in Reference to a Location. (A) CZECHII mouse's polar angles across the first 25 min of a session. Lines represent progression segments and dots represent lingering episodes. The path near the wall is shown in black and the path in the center—in gray. (B) The extraction of a sequence of passings and stops from a time-series of the mouse's polar angles during the first 2.5 min. The horizontal line denotes a specific polar location for which the sequence of visits is extracted, and the numerals printed within squares indicate the ordinal numbers of the visits, white squares for passings, and black—for stops. Only the path near the wall (in black) is used for scoring. The enumerated squares construct, from bottom to top, the column on the right, which depicts the sequence of passings and stops in the selected location.

Since most activity takes place at the periphery of the circular arena (see Methods), we moved to polar coordinates description of the smoothed path (with (0,0) at the center of the circular arena). As illustrated in Figure 1A, the polar projection of the mouse's path as a function of time was punctuated by stops (black dots) in an apparently sporadic manner. While the mouse's decision to stop at a specific location upon traversing it could be taken randomly we wanted to take a closer look at the possibility that it still depended on the history of visits to that location. For that purpose we first established a record of visits in reference to a location, classifying each visit as a stop or a passing through. We then studied jointly records for all locations, and calculated the probability of stopping during a specific visit to a location as a function of the ordinal number of that visit. A change in this probability across visits would have implied that the decision to stop was influenced by visiting history.

The procedure of establishing a record of visits in reference to a location is illustrated in Figure 1B: angular position 270° is represented by a straight line parallel to the x-axis. By following the line one can see that upon visiting this location the mouse did not stop in it during the first 3 visits, stopped in it during the 4^th^ and 5^th^ visit, and then again passed through it without stopping during the 6^th^ visit, etc. This sequence of discrete events, consisting of 3 successive *passings*, 2 *stop*s, 1 *passing*, and another 2 *stops*, is presented from bottom to top in the right column of Figure 1B. Similar sequences of passings and stops were obtained for all 120 locations defined by the grid superimposed on the periphery of the arena (see Methods).

**Figure 2:**
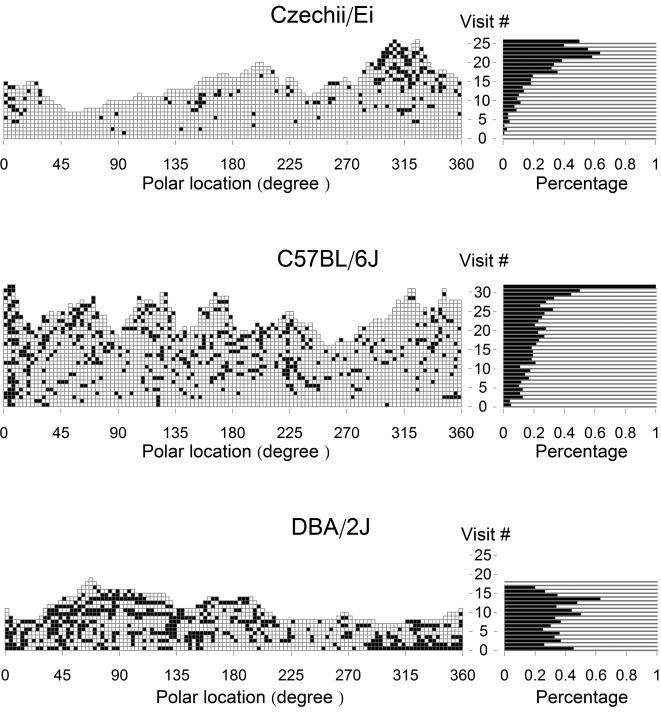
The History of Visits to Peripheral Locations in the Arena. (Left) History of visits to all peripheral locations during a 30-min session of 3 mice belonging to 3 different inbred strains. White squares represent passings, and black squares—stops. (Right) Probability of stopping as a function of the ordinal number of visits. Each horizontal bar represents the proportion of stops performed during the nth visit to a location, by summing up the stops and passings belonging to the corresponding row plotted in the left panel. The black portion of the bar represents the percentage of stops performed during the nth visit to all locations in which such visit occurred (the white portion represents the complementary percentage of passings). As illustrated, the probability of stopping increased as a function of the ordinal number of a visit in the CZECHII and C57 mice, and did not change in the DBA mouse.

The sequences of passings and stops obtained for all locations in 3 representative mouse-sessions are shown in the graphs of Figure 2, left panel. An overview of these graphs reveals that the stops appeared to be distributed evenly throughout the sequences in the DBA mouse, but occurred mostly during later visits in the CZECHII mouse. The increase in stopping frequency across visits was also present in the C57 mouse, but in a milder form. These tendencies appeared to characterize the 3 strains (Figure S2).

We estimated the probability (*p_n_*) of stopping during a visit to a location as a function of the ordinal number of that visit in the following way. With *V_n_* being the number of such *n*-th visits, *S_n_* out of the *V_n_* visits had been classified as stops. The proportion *P_n_ = S_n_/V_n_* was the desired estimator of the probability of interest *p_n_* (see Methods). As shown in the right panel of Figure 2, the probability of stopping increased as a function of the ordinal number of a visit in the CZECHII and C57 mice, and did not change in the DBA mouse. In other words, in these CZECHII and C57 mice, the decision to stop in a location was influenced by the number of previous visits paid to that location, whereas in the DBA mouse, visiting history did not affect this decision.

**Figure 3:**
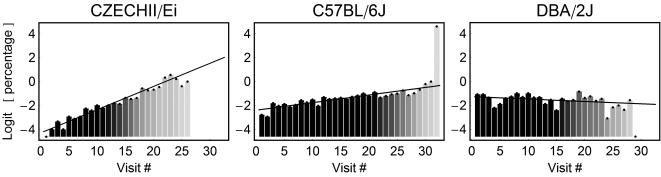
Rate of Change in the Probability of Stopping as a Function of Number of Visits. 3 examples of a linear regression fitted to the normalized probabilities of stopping data. The graphs are similar to the graphs in Figure 2, right panel. Each vertical bar represents the percentage of stops performed during the n^th^ visit to all locations in which such visit occurred. Gray level of bars denotes the weight assigned to the probability value used for the calculation of the linear regression. The data are transformed in order to allow the fitted regression to be linear (see Methods). The black line depicts the regression. The rate of change in the probability of stopping as a function of the ordinal number of a visit was indicated by the slope of the fitted linear function, which reflected a significantly positive trend in CZECHII and C57 mice, and no significant trend in the DBA mouse.

**Figure 4:**
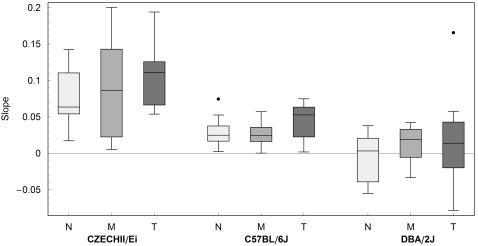
Rate of Change in the Probability of Stopping at a Location. Boxplot summaries of the rate of change in the probability of stopping at a location as a function of the number of previous visits to that location, in 3 strains and across 3 laboratories. Results obtained in NIDA (N), MPRC (M), and TAU (T) are shown, respectively, in light, medium, and dark gray. The trend of the rate of change in the probability of stopping at a location for CZECHII and C57 was significantly positive (p<.0001 and p = .009 respectively) while for the DBA it was not (p = .28).

To quantify the rate of change in the probability of stopping, we fitted a linear function of *n* to the logit-transformed *p_n_* in the form (Figure 3):
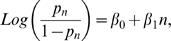
for each mouse. The estimated slopes for all 3 strains are presented in Figure 4. All mice of the CZECHII showed an increase in the probability of stopping as the number of visits increased, so did the trends of all mice of the C57 strain, though the trends were closer to 0. In contrast, DBA showed mixed trends, 21 increasing and 14 decreasing trends. See Figure 4 for the summary of the individual mice trends per each strain and laboratory. Pooling across laboratories using fixed model ANOVA we found that the trend for CZECHII and C57 was significantly positive (p<.0001 and p = .009 respectively) while for the DBA it was not (p = .28) (all results are deposited in the database of the Mouse Phenome Project, [15]).

Putting the result through a more stringent test for replicability, by using the mixed model ANOVA where laboratories were treated as random as well as their interaction with strains [16], we found that the difference in slopes across strains was highly statistically significant (p<.0001). Furthermore, 95% confidence interval for the slope for CZECHII was (.053, .132), for C57 is (−.001, .069) and for DBA was (−.026, .045) giving similar results to those of the fixed effect.

To rule out the possibility that changes in the probability of stopping reflect the level of activity of the animal per session, the Pearson Correlation Test was performed on Distance Traveled near the wall and the slope value obtained from each animal. The correlation was small, r = −.2 and not statistically significant at the .5 level.

**Figure 5:**
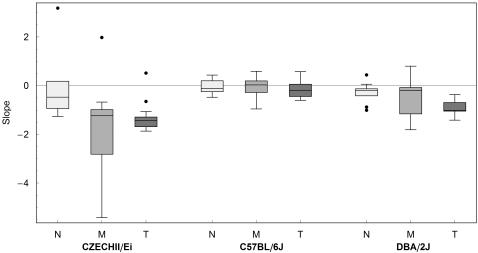
Rate of Change in the Frequency of Stopping across Time. Boxplot summaries of the rate of change in the frequency of stopping across time, in 3 strains tested simultaneously in 3 laboratories. Results, obtained from NIDA (N), MPRC (M), and TAU (T), are shown in light, medium, and dark gray, respectively. The slopes of all strains in all laboratories were either parallel to the x–axis or negative, implying that the frequency of stopping did not increase across the session.

The visiting sequences used for the computation of the slopes of regression described the order of visits to the same location; they did not provide the time of the visits' occurrence. The increase in the probability of stopping at locations could, therefore, merely reflect an increase in the frequency of stopping across the session. To examine this possibility we scored the number of stops per sliding time window (3-min time bins with an overlap of 1 min) across the session, fitted a linear regression to the obtained values, and computed the slope of the line. As can be seen in Figure 5, the slopes of all strains in all laboratories were either parallel to the x–axis or negative, implying that the frequency of stopping did not increase across the session. Therefore, changes in the frequency of stopping across time could not explain the change in the probability of stopping at a location with increasing number of visits.

Having ruled out the possibility that the frequency of stopping increases across time, and having shown in the previous section the replicability of the results in 3 laboratories, we concluded that the rate of change in the probability of stopping as a function of visiting history was a reliable measure of mouse locational memory in the open-field.

### Dynamics of Stopping in Specific Locations

**Figure 6:**
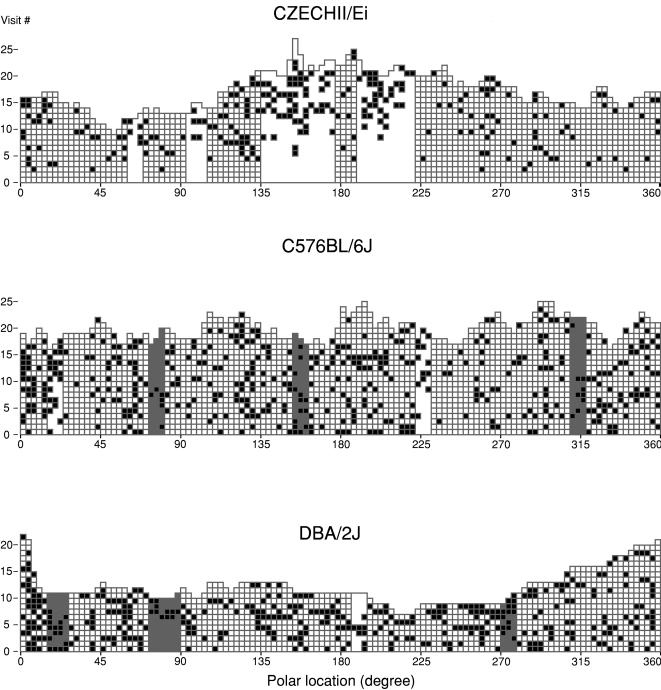
Places Marked by an Increasing or Decreasing Probability of Stopping. History of visits to all peripheral locations across 30-min sessions of 3 mice. White squares: passings, black squares: stops. White stripes mark places in which the probability of stopping increases; gray stripes mark places in which the probability of stopping decreases. The CZECHII mouse was characterized by having only places where the probability of stopping increased or stayed unchanged; in contrast, the C57 and DBA mice were characterized by having all 3 types of places.

The changes in the probability of stopping (Figure 4) were computed by pooling the data across all locations at the periphery. Therefore, the results presented so far applied to all locations in a general way, ignoring changes at specific locations. Further investigation of the data collected in TAU revealed 3 types of locations: those in which the probability of stopping increased, those in which it decreased, and those in which it stayed unchanged (see Methods). The locations showing an increase appeared in clusters and so did the locations showing a decrease (see Figure 6 and S2). In order to distinguish between the arbitrarily defined single locations, and their clusters, which were revealed by our analysis, we termed the clusters places (it should be noted that the minimal number of locations in a cluster is 3, reflecting our measurement resolution; see Methods).

As shown in Figure 6 in 3 examples, the CZECHII mouse was characterized by having only places where the probability of stopping increased or stayed unchanged; in contrast, the C57 and DBA mice were characterized by having all 3 types of places; finally, the DBA mouse was characterized by having the highest number of places where the probability of stopping stayed unchanged. Those strain differences prevailed in all the mice tested in TAU (see Figure S2 for graphs of all mice).

## Discussion

### Intrinsic Constraints on Stopping Behavior Imply Locational Memory

In this study we show that in the open field, visiting history to a location influences stopping behavior in that location; the magnitude of this influence is strain specific. In 2 out of 3 examined strains, the higher the ordinal number of a visit to a location, the higher is the probability of stopping in that location. In the third strain, the ordinal number of a visit to a location appears to be irrelevant for the decision whether to pass through the location or stop in it. In the strains that show increased probability of stopping with consecutive visits to a location, this is not due to a general tendency of the mice to stop more frequently with time. On the contrary—the tendency to stop either decreases or stays unchanged across the session in all mice and strains (Figure 5). Because the phenomenon depends on the ordinal number of visits, it implies some type of memory, and because it describes behavior in specific locations, it is spatial. Taken together, it indicates a locational memory.

### Locational Memory and Spatial Memory

Future studies would tell us to what extent locational memory utilizes the various sensory modalities. Hippocampus-guided spatial memory is, for example, commonly demonstrated by showing that manipulation of distal visual cues is followed by corresponding adjustments in the animal's spatial orientation [17]. In real life situations, spatial orientation may also be supported by the processing of cues belonging to the other sensory modalities, including proprioception derived from self movement [18], yet the term spatial memory became mainly identified with visual processing. The locational memory highlighted in the present study implies spatial recognition and familiarity, and therefore also reflects spatial memory, but the particular contribution of each of the sensory modalities to the mouse's orientation is not known. Support for visual guidance by distal cues is indicated by the consistency of our results with those obtained for visually guided tests of spatial memory in 2 of the strains (CZECHII mice have not been tested yet for spatial memory). Thus, as with locational memory, good spatial memory is exhibited in C57 in various spatial tasks [9], [19]–[21]. The absence of a locational memory in DBA/2 mice similarly corresponds to the lack of spatial memory reported in most studies performed on this strain [7]–[9], known to suffer from hippocampal dysfunction [22]–[24]. These parallel findings support the hypothesis that the memory described in this paper is also guided visually. The hypothesis that a change in the probability of stopping in locations across visits is mediated by the accumulation of olfactory cues, which are in turn accumulated across visits is untenable, as it would require a mechanism explaining why scent accumulation has no influence on stopping in DBA/2 (Figure 6, lower panel; Fig S2), a strain gifted with a more sensitive olfactory sense than C57 [25],[26], does influence stopping, but in 2 opposite ways, in C57 mice (Figure 6, middle panel; Fig S2), and only increases stopping in the CZECHII mice (Figure 6, upper panel; Fig S2). Estimating locational memory in open field behavior recorded in full darkness would tell us to what extent this construct is supported by information derived from self movement. Finally, dependence on the hippocampus can be investigated by using lesions or temporary inactivation of this structure, and the role played by memory on this phenomenon, although not specific to spatial memory only, can be investigated by using pharmacological disruption that is predictive of memory loss. Whatever the underlying mechanisms, locational memory, which has been shown to be strain-specific can now be compared across strains and preparations.

### The Relationship between Locational Memory and the Level of Activity

Since our measure is based on locomotor behavior, there is a concern that this measure is influenced by the animal's level of activity. To rule out this possibility we examined the correlation between distance traveled per mouse-session and the corresponding rate of change in the probability of stopping as a function of the number of visits. The correlation was small and not statistically significant (r = −.2, p<.05), implying that within the range of values obtained in this study, the level of activity does not influence our measure.

Examination of pharmacological preparations exhibiting hyperactivity (e.g., [27],[28]) could further elucidate the issue of the influence of activity on the measure of locational memory. In 3 previously performed studies on the effect of dopamine-stimulants on locomotor behavior in general, and on stopping in locations in particular, all 3 drugs induced hyperactivity, but had 3 distinct effects on stopping in locations. (+)-amphetamine-induced hyperactivity was associated with a consolidation of stereotypic stopping in a limited number of locations in a relatively fixed order [29]; quinpirole- induced hyperactivity was associated with the performance of stopping in 2 fixed and several varying locations between them [30]; and apomorphine-induced hyperactivity was dissociated from stopping in fixed locations, showing no organization in relation to the environment [31]. Since under the influence of the first 2 drugs the probability of stopping in specific locations increases, locational memory is implied, and our measure would have reflected it. The absence of an increase in the probability of stopping under the influence of the 3^rd^ drug would have resulted in a near-zero rate of change in the probability of stopping implying no locational memory.

### Newly Derived versus Classical Inbred Strains

CZECHII mice show a significantly higher rate of change in stopping probability than C57, implying even better spatial abilities. Some researchers consider the behavior observed in classic inbred strains to be dull and “degenerate” [32], whereas wild-mouse behavior is expected to exceed the behavior of these strains [33],[34] in terms of repertoire richness [35], and magnitude of parameters [11],[36]. CZECHII mice are a relatively new wild-derived strain, perhaps less affected by the domestication process; the enhanced spatial performance of this strain could be ascribed to its relative wildness.

### A Bottom-Up Approach to Higher Cognition-Related Constructs

The bottom-up approach employed by us aims at revealing higher-level phenomena, as they emerge out of low-level kinematic properties. In the present study, assigning visiting records to locations, and characterizing the sequences constituting these records, reveals locational memory. This phenomenon adds up to a list, reviewed below, of previously described higher-level phenomena also uncovered by the bottom-up approach.

Noting where rats stop, and for how long, highlighted the home-base-the s most preferred place in the arena [3]. Using this place as a reference for measuring kinematic properties of the rat's trajectory revealed several features of the rat's operational world. Partitioning the rats trajectory into roundtrips performed from the home-base highlighted a gradual lengthening of these roundtrips. This lengthening was correlated with an increasing amount of exposure to the arena. It defined, therefore, the animal's increasing familiarity with the environment [5]. A high level of familiarity ( =  exposure) was also indicated by a reversal of speed differences in relation to the home-base: in a novel environment, the outbound portion of a trip was characterized by lower speeds, and the inbound portion–by higher speeds; in a well-trodden environment the speed difference was shown to be reversed. These speed differences together with the amount of exposure defined “inbound” and “outbound” directions from the rat's point of view [5],[37],[38]. In still another study, the ordinal number of a stop within a roundtrip was found to determine the magnitude of a rat's attraction to the home-base; as the ordinal number of the stop increased, the attraction, expressed as the probability of returning home after stopping, increased as well [6]. Absence of speed differences between inbound and outbound portions were used to infer navigation impairments in hippocampectomized rats [39],[40].

In summary, the dynamics of roundtrip length and of inbound/outbound speed differences were used to define familiarity; the ordinal number of a stop within a trip was used to estimate home-base attraction; and the dynamics of stopping as a function of the ordinal number of visits to locations was used in the present study to estimate spatial memory. The increasing tendency to stop in well-trodden places, in the sense offered by von Uexkull [41], reflects the consolidation of a spatial habit: repeated visits to a location are accompanied by an increasing tendency to stop in that location, culminating in turning it into a relatively stable spatial attractor ([42]; or, in the case of a decreasing tendency to stop, a repeller).

### Classifying Locations by Their Level of Attraction

A gradual increase or decrease in the probability of stopping along trodden paths reflects respectively an increasing attraction or an increasing repulsion to a location. Whereas the CZECHII mice developed places of attraction and no places of repulsion (Figures 6 and S2), the C57 mice (and to an extent also the DBA mice) developed both types. To test the statistical significance of these apparent regularities in single locations, it would be necessary, however, to extend the duration of sessions in order to obtain a much larger number of visits per location.

### An Improved Analytical Model of the Kinematic Structure of Rodent Exploratory Behavior

A simple analytical model of rodent exploratory behavior simulated the observations made on real rat open-field behavior [5] by using a sim-rat [37]. The sim-rat increases excursion distance from home-base as a linear function of two system parameters, one governing the rate of motivation loss during movement away from the home-base, and the other the rate of (location-specific) familiarization. The sim-rat's velocity pattern is correlated with the familiarity with places, changing gradually from slow-outbound–fast-inbound, to fast-outbound–slow-inbound. It had been concluded in that analytical study that one shortcoming of the model was that the sim-rat moved continuously, while the movement pattern of a real animal includes stops. It has been further suggested that a comprehensive model of exploratory behavior should include a stochastic component accounting for the stops. The measured changes in the probability of stopping along well-trodden paths specify this stochastic component.

### A High Throughput Test of Spatially Guided Behavior in a Less Stressful Environment

The test commonly used for the estimation of spatial memory is the Morris water maze [43]. Other tests include, e.g., the radial arm maze [44], the modified hole board test [45], and the spatial open field [21]. The pros and cons of these and other setups have been discussed elsewhere, and it has been suggested that no single task can reveal the full richness of spatially guided behavior (e.g., [36]). The present study supplements the arsenal of already available tools with a new measure and a new high throughput test of spatially guided behavior conducted in a single session in a large, dry, and empty open field arena.

## Methods

The data for this study were collected in a study conducted simultaneously in 3 laboratories: The National Institute on Drug Abuse (NIDA), Baltimore; Maryland Psychiatric Research Center (MPRC), Baltimore; and Tel Aviv University (TAU). These data are stored in a publicly available database (http://www.tau.ac.il/ilan99/see/help), and have already been used in previous studies [11]–[14],[46],[47]. The study included 10 inbred mouse strains and was part of the Mouse Phenome Database project [15]. In this work, we used only the data of the C57BL/6J, DBA/2J and CZECHII/Ei strains.

The experimental and housing protocols were identical for all the above studies, and were described in detail elsewhere [13]. Here we repeat the main points.

### Animals

9–14 week old C57BL/6J (C57), DBA/2J (DBA) and CZECHII/Ei (CZECHII) males shipped from Jackson Laboratories. The sample sizes were 12 per C57BL/6J group in each laboratory, 12 per DBA/2J group in each laboratory, and 6 per CZECHII/Ei group in NIDA, 8 in MPRC, and 12 in TAU.

### Housing

Animals were kept in a 12∶12 reversed light cycle (Light: 8:00 p.m.–8:00 a.m.), and were housed 2–4 per cage under standard conditions of 22°C room temperature and water and food *ad libitum*. The animals were housed in their room for at least 2 weeks before the start of the experiment. All animals were maintained in facilities fully accredited by the American Association for the Accreditation of Laboratory Animal Care (AAALAC, MPRC and NIDA) or by NIH Animal Welfare Assurance Number A5010-01 (TAU). The studies were conducted at all 3 locations in accordance with the Guide for Care and Use of Laboratory Animals provided by the NIH.

### Experimental Procedure

The arenas were 250 cm diameter (TAU, NIDA) and 210 cm diameter (MPRC) circular areas with a non-porous gray floor and a 50-cm high, primer gray painted, continuous wall. Several landmarks of various shapes and sizes were attached in different locations to the arena wall and to the walls of the room where the arena was located. In particular, one wall of the room was mostly covered in black, and a large dark rectangle of 60×80 cm was painted on each of the 2 adjacent walls. The arena was illuminated with two 40-W neon bulbs on the ceiling, above the center of the arena.

The experiments were conducted during the dark part of the cycle, 1–2 hours after its onset. Each experimental animal was brought from its housing room to the arena in a small opaque box, and placed within it (in a standardized location, near the wall) while still in the box. After 20 seconds the box was lifted, and a 30-min session began. The arena was recorded using a resolution of 25 (TAU) or 30 (MPRC, NIDA) samples per second and approximately 1 cm. The animal's movement was tracked using Noldus EthoVision automated tracking system [48].

### Data Analysis

The raw data obtained from the tracking system were smoothed using a specialized algorithm implemented in the stand-alone program “SEE Path Smoother” [13],[49]. This procedure produces reliable estimates of momentary speeds during motion (momentary speeds during arrests were defined as zero).

As was previously shown, rodent locomotor behavior consists of two distinct modes of motion—progression segments and lingering episodes [2],[6]. During progression segments, the animals traverse relatively large distances attaining relatively high speeds. During lingering episodes the animals stop and perform scanning movements, while staying in a circumscribed neighborhood. Segmentation of the smoothed path into progression segments and lingering episodes was done using the EM algorithm [50] with a two-gaussians mixture model. Stand-alone user-friendly software for smoothing (SEE Path Smoother) and for segmentation (SEE Path Segmentor) can be downloaded at http://www.tau.ac.il/ilan99/see/help.

### Defining Sequences of Visits

**Figure 7:**
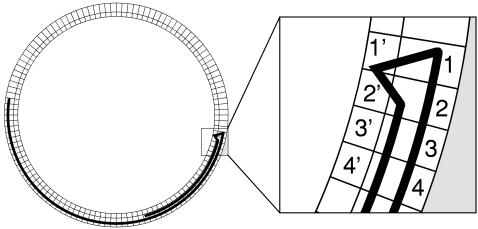
Schematic Illustration of the Partitioning of the Path into Visits. The circle represents the open-field arena with the superimposed grid, consisting of 2 layers of locations. The outer layer was used to define visits to locations, and the inner layer was used to define additional adjacent locations used to avoid false fractionation of visits to outer-layer locations. Insert: outer-layer locations are indexed by numerals, and their corresponding adjacent inner-layer locations are indexed by corresponding numerals with an apostrophe. A black line represents the path traced by the mouse. In this example, 2 visits were scored in locations 3 and 4, 1 visit in location 2, and no visit in location 1.

Because the vast majority of locomotor behavior is performed along the wall [12],[14] we focused on the path traced by the mouse near it. To quantize the path into sequences of visits to locations, we first schematically superimposed a circular grid consisting of 7×10 cm rectangles on the periphery of the arena. We then partitioned the path traversed by the animal near the wall into a sequence of visits to the locations defined by the grid rectangles. A visit to a location started when the mouse entered the location and ended when it left the location. Because the locations had been defined in an arbitrary way, small insignificant trespassing of the path into adjacent rectangles would have been considered as visits. Therefore, 2 successive visits to the same rectangle were considered as such only if the mouse reached a “long enough” distance from that rectangle between the 2 visits. A “long enough” distance was defined as the distance necessary in order to enter a location that is not adjacent to the original location (see Figure 7). In order to fully surround each location with adjacent locations, an inner-layer of 7×7 cm rectangles was added (the length of the side of the inner-layer rectangles was set to be the same as the width of the outer-layer rectangles).

In the next stage of the analysis, all visits were categorized as either stops (visits containing a lingering episode) or passings (visits that did not contain a lingering episode).

### Statistical Methods

#### Computation of the slopes of linear regression fitted to the normalized probabilities of stopping

We estimated the probability (*p_n_)* of stopping during a visit to a location as a function of the ordinal number of that visit in the following way. Visits at locations are classified according to whether they constitute an *n*-th visit to a location or not. Let *V_n_* be the number of such *n*-th visits (obviously each *n*-th visit is at a different location); such *V_n_* can be calculated for any *n*. Out of the *V_n_ n*-th visits *S_n_* have been classified as stops. The proportion *P_n_ = S_n_/V_n_* is the desired estimator of the probability of interest *p_n_*.

As often happens when studying the dependence of probabilities on explanatory variables, the dependency of *p_n_* on *n* seems to follow a logistic model. Namely, we fit a linear function of *n* to the logit-transformed *p_n_* in the form
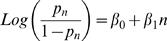



In this model β_0_ is the intercept and β_1_ is the slope: β_1_ captures the change in the logarithm of the odds *p_n_*/(1*−p_n_*) for a stop, from the *n*-th visit to the ***(*** *n*+1)-th visit (or equivalently the odds changes gradually from one visit to the next).

Since the variance of *P_n_* as an estimator of *p_n_* increases as *V_n_* decreases, being based on a smaller sample, and the latter obviously happens because during late visits the mouse visited increasingly fewer locations, the fitting of the logistic model is based on weighted regression with weights proportional to *V_n_*.

#### Comparing endpoint results between strains and across laboratories

In order to assess the discrimination between strains and the replicability across laboratories of slopes of logistic regression (see the results section below), we used the linear mixed effects ANOVA model [51],[52]. In this model, the strain was considered as a fixed factor while the effect of laboratory was considered as random. This means that we thought of the laboratory effect as being drawn from the population of all possible laboratories effects. The interaction between strain and laboratory was considered random as well. Thus, a significant strain difference yielded by the Mixed effects Model ANOVA can be regarded as replicable across laboratories. This approach is more conservative than the widely used linear fixed effects model ANOVA: if a difference between two strains was found to be significant under the mixed model, it will be significant under the fixed effects model as well, but the opposite is not necessarily true [16].

One DBA/2 mouse who did not travel along the whole circumference of the arena even once during the session, was excluded from the analysis.

#### Classifying locations by the change in the probability of stopping

To investigate changes in the probability of stopping at specific locations, one would have to record many more visits per location than can be collected during a 30-min session. Therefore, we increased sample sizes by pooling the visits paid to 3 adjacent locations at a time, moving along the periphery of the arena with a step of 1 location. To examine the change in the probability of stopping within each group of selected locations we divided each sequence of visits to these locations into 2 halves (Figure S1). When the number of visits was uneven, the visit in the middle of the sequence was excluded from the analysis. We then compared the number of stops and the number of passings in the first half, to their sums in the second half and classified the locations according to the change in the probability of stopping in them. In order to determine whether there was a significant change in the number of stops we used the Fisher Exact Test.

## Supporting Information

Figure S1 Classification of Visited Locations. (A) The history of visits to peripheral locations during a 30-min session. White squares represent passings, and black squares-stops. Each sequence of visits to a location is divided into two halves (Red line). (B) Locations with a significant increase in the amount of stops are shown in gray. (C) Locations with a significant increase in the amount of stopping are shown along the periphery of the arena in black. The path traced by the animal across the session is shown in gray.
(1.04 MB TIF)

Figure S2 Places Marked by an Increasing or Decreasing Probability of Stopping. History of visits to all peripheral locations across 30-min sessions of all the mice studied in TAU. White squares: passings, black squares: stops. White stripes mark places in which the probability of stopping increases; gray stripes mark places in which the probability of stopping decreases. The CZECHII mice were characterized by having only places where the probability of stopping increased or stayed unchanged (with an exception of one place in one mouse); in contrast, the C57 and DBA mice were characterized by having all 3 types of places.
(3.88 MB TIF)
