# Supplementary figures and images for: Mouse Cognition-Related Behavior in the Open-Field: Emergence of Places of Attraction
Source: PLoS Comput Biol. 2008 Feb 29;4(2):e1000027. doi: 10.1371/journal.pcbi.1000027 (PMC2265485; doi:10.1371/journal.pcbi.1000027)

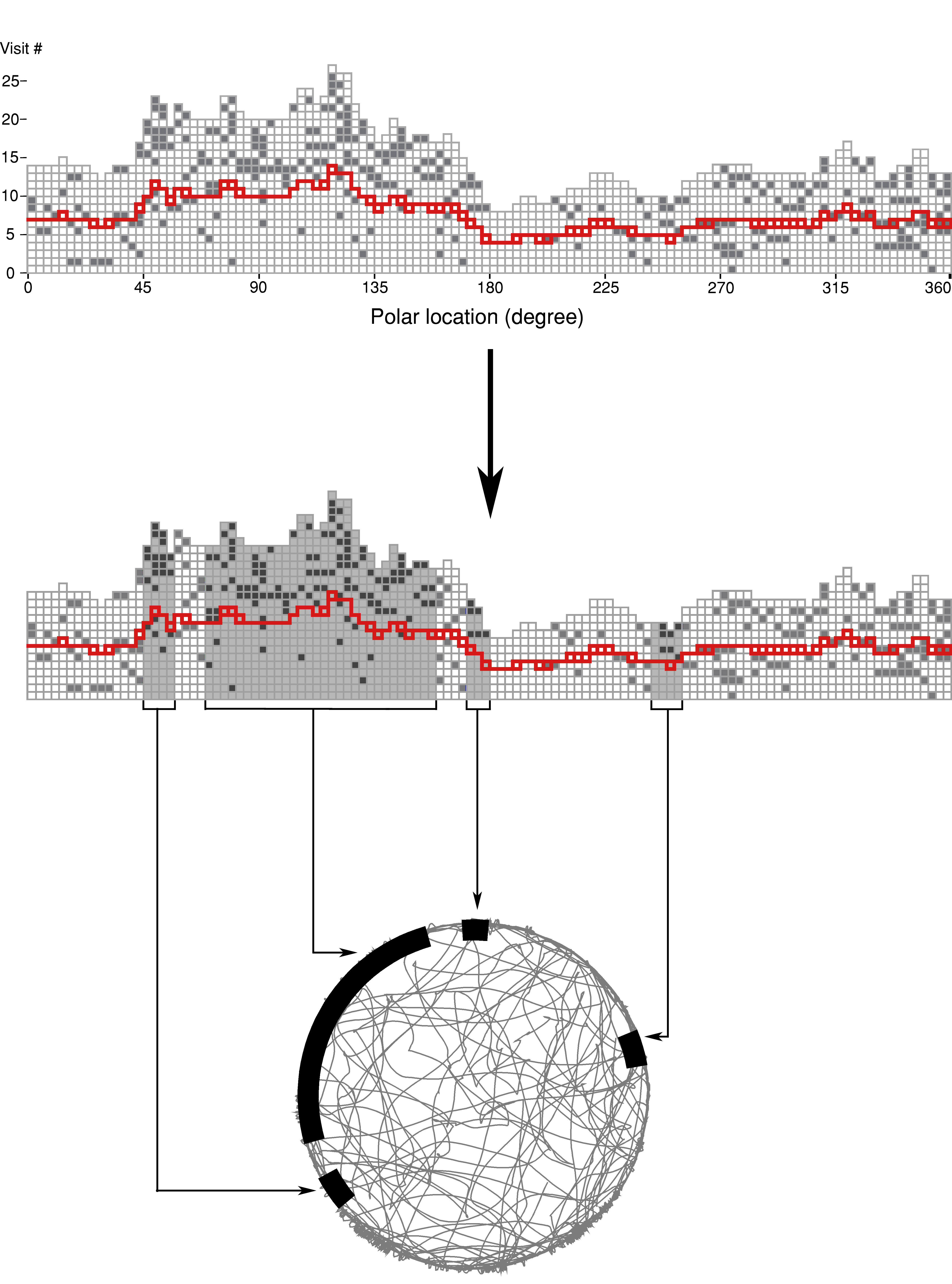

Supplement: Figure S1 — Classification of Visited Locations. (A) The history of visits to peripheral locations during a 30-min session. White squares represent passings, and black squares-stops. Each sequence of visits to a location is divided into two halves (Red line). (B) Locations with a significant increase in the amount of stops are shown in gray. (C) Locations with a significant increase in the amount of stopping are shown along the periphery of the arena in black. The path traced by the animal across the session is shown in gray. (1.04 MB TIF) [file pcbi.1000027.s001.tif]
